# Oligonucleotide Synthesis Errors Are a Source of Untoward Variation in HDR-Mediated Gene Editing

**DOI:** 10.3390/genes17070729

**Published:** 2026-06-24

**Authors:** Stacia K. Wyman, Zulema Romero, Seok-Jin Heo, Marian Navarrete, Netravathi Krishnappa, Donald B. Kohn, David I. K. Martin, Mark C. Walters, Dario Boffelli

**Affiliations:** 1 Innovative Genomics Institute, University of California, Berkeley, CA 94720, USA; staciakwyman@gmail.com (S.K.W.); netrak@berkeley.edu (N.K.); 2 Departments of Microbiology, Immunology, and Molecular Genetics, University of California, Los Angeles, CA 90095, USA; zulemar@ucla.edu (Z.R.); marian977@g.ucla.edu (M.N.); dkohn1@mednet.ucla.edu (D.B.K.); 3 Laboratory for Genomics Research, Department of Biochemistry and Biophysics, University of California, San Francisco, CA 94158, USA; seok-jin.heo@ucsf.edu; 4 UCSF Benioff Children’s Hospital Oakland, Oakland, CA 94609, USA; dikm9wjm@gmail.com (D.I.K.M.); mark.walters@ucsf.edu (M.C.W.); 5 Department of Pediatrics, Cedars-Sinai Guerin Children’s, Los Angeles, CA 90069, USA

**Keywords:** gene editing, sickle cell disease, single-stranded oligonucleotide, DNA synthesis

## Abstract

**Background/Objectives:** Single-stranded oligonucleotides (ssODNs) are used as donor templates for therapeutic gene editing by CRISPR-Cas9 cleavage and homology-directed repair (HDR). Although ssODN sequence fidelity is critical to the safety and efficacy of editing, standard quality control methods cannot resolve individual nucleotide errors. **Methods:** We performed deep sequencing of ssODNs from three manufacturers and amplicons from edited hematopoietic stem/progenitor cells. **Results:** We find that synthesis errors are present in all ssODNs tested at rates that vary more than two-fold among manufacturers, at positions that are dependent on sequence context. These synthesis errors are propagated into the genome by HDR at frequencies proportional to their abundance in the ssODN. In our sickle cell mutation correction protocol, the most prevalent SNEs are predicted to produce benign β-globin variants, while the less frequent frameshift deletions are predicted to generate β-thalassemia-like alleles. **Conclusions:** Current quality control standards are insufficient to detect these errors, and deep sequencing of ssODNs should be incorporated into regulatory submissions for clinical gene editing programs.

## 1. Introduction

Single-nucleotide mutations that cause genetic disease can be corrected by gene editing protocols in which endogenous cellular DNA repair mechanisms make use of an exogenous donor template to repair a double-stranded break (DSB) created by the Cas9 endonuclease at or near to the site of the mutation. ssODNs are attractive templates for HDR because they can readily be synthesized with a specified sequence and length, and avoid production costs and toxicity inherent in viral DNA templates [1]. Although oligonucleotide synthesis is a mature technology, the quality of the synthesis product is routinely assessed only by liquid chromatography, gel electrophoresis, and mass spectrometry, with sequence fidelity not directly confirmed. The sequence fidelity of chemically synthesized oligonucleotides has been characterized in a few previous studies. Filges et al. showed that deletions predominate over substitutions, with error profiles that vary by manufacturer, purity grade, batch, and sequence context [2]. Further studies demonstrated that substitution errors, particularly G-to-A transitions, are strongly influenced by the capping step of phosphoramidite synthesis, and proposed modifications to synthesis chemistry that reduce their frequency [3,4]. Collectively, these studies establish that synthesis errors in ssODNs are reproducible and sequence-context-dependent but share the critical limitation that their sequencing approaches required oligonucleotides specifically designed to enable library construction. These methods are therefore not applicable to the characterization of ssODNs whose sequences are fixed by therapeutic design, as is the case in clinical gene editing protocols. Furthermore, none of these studies addressed whether synthesis errors are propagated into the genome when an ssODN is used as a donor template for HDR, leaving the consequences of synthesis errors for therapeutic applications uncharacterized. A sensitive and broadly applicable means of assessing sequence fidelity is needed.

HDR-mediated gene editing can be templated by several classes of donors: double-stranded DNA donors, including plasmids, PCR products, and double-stranded oligodeoxynucleotides [5]; viral donors, most prominently recombinant adeno-associated virus serotype 6 [6]; and single-stranded donors, comprising ssODNs and longer single-stranded DNA [7,8]. The work reported here concerns the single-stranded class, although the chemical-synthesis error we describe is a property of oligonucleotide synthesis itself and applies to any chemically synthesized donor regardless of length or strandedness. Two features distinguish our approach from the prior digital-sequencing studies of synthesis fidelity: it resolves the sequence of any ssODN whose sequence is fixed by therapeutic design, using standard library-preparation reagents rather than the engineered constructs that previous methods required, and it establishes whether synthesis errors are carried into the genome during HDR, the question that bears directly on the safety of an edited cell product.

We developed a gene editing protocol to correct the mutation in the human β-globin gene (*HBB*) that causes Sickle Cell Disease (SCD) [7,8]. Cas9 is directed by a single guide RNA (sgRNA) to introduce a double-stranded break in *HBB* 18 base pairs (bp) from the SCD mutation in autologous hematopoietic stem and progenitor cells (HSPCs). The 168 bp ssODN has a sequence complementary to the region of *HBB* encompassing the SCD mutation, restoring an adenine base to correct the SCD mutation when used as a donor template for HDR. Clinical application of this protocol, and of any protocol aiming to correct a mutation using an ssODN, requires a tractable means to validate the identity and sequence of the ssODN and to understand what, if any, sequence changes might accompany correction of the mutation. We have addressed this need by creating deep sequencing libraries from oligonucleotides and corroborating apparent sequence errors by showing that they are propagated into the genome by HDR. A preliminary account of this work was presented in abstract form [9].

## 2. Materials and Methods

### 2.1. ssODN Library Preparation and Sequencing

ssODN sequencing libraries were constructed using the Claret Bioscience, Scotts Valley, CA, USA, SRSLY ssODN library preparation kit and IDT, Coralville, IA, USA, xGen RNA Library Preparation Kit following the manufacturer’s instructions. Libraries were sequenced using Illumina 150 bp paired end reads to a minimum depth of 3 M reads. Each ssODN was used to build a single sequencing library; after alignment and, for the SRSLY libraries, UMI-based deduplication, each retained read is an independent observation of a molecule from the synthesized pool (a mean of approximately 150,000 aligned reads per ssODN), so per-position error frequencies are estimated from that number of independent molecules per manufacturer.

Sequenced reads were trimmed and merged to single reads using bbmerge version 39.0.0, then aligned to the reference ssODN using bowtie2 version 2.4.5. Where UMI’s were used, reads were deduplicated using UMI-tools version 1.1.2. SNEs, insertions and deletions were then quantified using samtools version 1.15 and custom Python 3.10 scripts (see flow chart, Supplementary Figure S1). bbmerge was run in strict mode and providing the adapter sequence; bowtie2 was run with the following parameters: -L 30 -N 1 -D 20 -R 3. Before quantification, 8 bases were trimmed from each read end to exclude library-preparation artifacts that accumulate at fragment ends (Supplementary Figure S2), and positions were retained only above a minimum aligned depth. The control against sequencing or PCR artifact is empirical rather than computational: positions with elevated error rates in the directly sequenced ssODN reappear at proportional frequency in HDR-derived genomic amplicons but are absent from unedited reads and from genomic positions outside the HDR tract, a position-specific and HDR-restricted concordance that sequencing or amplification error cannot produce.

### 2.2. Editing of HSPCs

Editing of HSPCs with the three ssODNs was carried out as described Dewitt et al. [7] and Magis et al. [8]. Editing used wild-type Streptococcus pyogenes Cas9 from the Integrative Genomics Institute, Berkeley, CA, USA, delivered as a ribonucleoprotein complex with a chemically synthesized single guide RNA [7,8].

### 2.3. Amplicon Sequencing

HBB amplicon generation and sequencing were carried out as described in Dewitt et al. [7] and Magis et al. [8].

### 2.4. Sequence Data Analysis

Analysis of sequencing data for direct ssODN sequencing was performed as described in Supplementary Figure S1. Rates of incorporation into genomic DNA were calculated using the amplicon sequencing data. The mismatches and deletions from the read alignment were tallied for all reads to obtain the rate of SNEs for an individual sample. SNE frequency across samples was then compiled together for each ssODN and plotted as boxplots. Each box represents an individual position around the cut site in the sequenced amplicon. Deletions were counted on the first (left hand) nucleotide position of the deletion so that they were counted just once per deletion (versus in each position that the deletion spans).

### 2.5. Nucleotide Context Enrichment Analysis

For each manufacturer, positions were ranked by SNE error rate and the top 20% were designated high-error positions. Mononucleotide and dinucleotide frequencies among high-error positions were compared to their background frequencies in the full ssODN sequence using one-sided binomial tests (alternative = “greater”). *p*-values were adjusted for multiple testing using the Benjamini–Hochberg procedure. Fold enrichment was calculated as the ratio of observed to background proportion; confidence intervals are derived from the lower bound of the exact binomial confidence interval for the observed proportion, scaled by the background proportion. All analyses were performed in R 4.5.2.

The top-20% cutoff was chosen to balance statistical power against specificity. At this threshold each manufacturer contributes approximately ten high-error positions (50 positions multiplied by 0.20), enough to support the binomial test while remaining selective for genuinely elevated positions. A threshold sweep from 5% to 30% in 5% steps, performed per manufacturer and at two significance levels (*p*_adj_ < 0.05 and *p*_adj_ < 0.10), supports this choice: below 10%, only three to five positions pass the cutoff and the test has little power; above 25%, the selected set is diluted by positions near background and the observed proportions regress toward the background composition. The number of significantly enriched dinucleotide contexts plateaus near 20% for manufacturers B and C and shows the same, weaker pattern for manufacturer A, whose globally higher error rates raise its 80th-percentile cutoff (0.0259, versus 0.0115 for B and 0.00334 for C) and make relative enrichment harder to resolve. Confidence intervals are one-sided lower bounds: the lower limit of the exact binomial interval for the observed proportion, scaled by the background proportion, so that an interval whose lower bound exceeds one indicates enrichment that remains significant at the stated level after Benjamini–Hochberg correction.

## 3. Results

**Figure 1:**
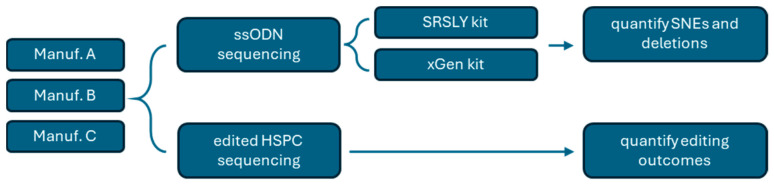
Schematic of sequencing strategy for three ssODNs, and of the SCD locus following editing of HSPC with each of the ssODNs. SNEs and deletions in the ssODNs were compared to editing outcomes.

We used three different vendors to manufacture the ssODN. The three vendors are commonly used commercial suppliers of research- and clinical-grade therapeutic ssODNs, chosen for their ability to produce oligonucleotide of the required purity; one of them (manufacturer C) provides GMP-grade oligonucleotide synthesis. While the purity of an ssODN was determined by the manufacturer using Ion Pairing HPLC and SDS-PAGE, and its identity was evaluated by liquid chromatography/electrospray ionization mass spectrometry, neither method confirms the specific nucleotide sequence of an ssODN or the absence of synthesis errors. To establish a method to quantify ssODN sequence fidelity and determine the best practices, we performed very deep sequencing of the ssODNs. We prepared sequencing libraries (Figure 1) using two different library preparation kits that are optimized for ligating adapters to single-stranded DNA: the SRSLY PicoPlus kit from Claret Bioscience which uses unique molecular identifiers (UMIs) to combat PCR bias, and the xGen kit from IDT (Commercial Park, IA, USA) which does not use UMIs. Each ssODN was sequenced with a minimum of three million 150 bp paired-end reads. Following alignment to our reference ssODN sequence and read deduplication using the UMIs (for the SRSLY kit), we achieved an average minimum depth of 150,000 aligned reads (Figure S1).

**Figure 2:**
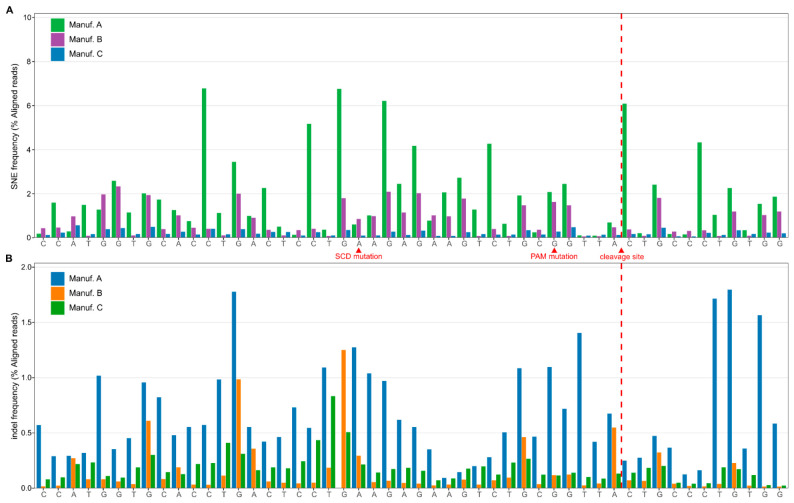
SNE (**A**) and indel (**B**) frequency in the three ssODNs, determined by direct sequencing. The *x*-axis shows a 50 bp window containing the Cas9 cleavage site specified by the guide RNA (vertical dashed red line) is shown, the PAM motif, and the SCD mutation; similar SNE levels were observed outside this region (see Supplementary Figure S2). The *y*-axis shows SNE frequency calculated as the percentage of aligned reads that contains an error. The Cas9 cleavage site (vertical dashed red line), the PAM motif mutation, and the SCD mutation are annotated on the *x*-axis.

Analysis of the data aligned to the ssODN reference revealed single nucleotide errors (SNEs) and short deletions at a much higher frequency than would be expected due to sequencing errors or PCR bias alone. Notably, the frequency of both types of error varied dramatically by manufacturer, with the SSODN from manufacturer A typically having over two-fold more errors than the ssODNs from manufacturers B and C. SNEs were found to occur more frequently at specific positions within the ssODN sequence across all three vendor-sourced ssODNs (Figure 2A and Supplementary Figure S2A), while small deletions (typically 1–2 bp in length) were more uniformly distributed across the sequence (Figure 2B and Supplementary Figure S2B). The rate of errors at individual positions was as high as 9% for SNEs and 3% for deletions, with the highest abundance seen in the ssODN from manufacturer A for SNEs and manufacturer C for deletions. The SNEs do not involve specific nucleotide conversion: for example, a site where a C nucleotide was specified might be modified primarily to a T, but also have changes to G or A.

**Figure 3:**
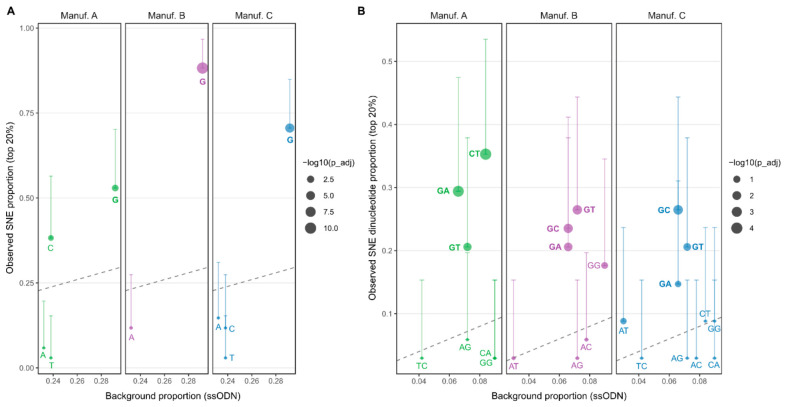
Nucleotide context enrichment at high-error positions in the ssODN. (**A**) Mononucleotide fold enrichment: individual bases significantly over-represented (BH-adjusted binomial test, *p*_adj_ < 0.05) among the top 20% highest-error positions relative to background base composition of the ssODN. (**B**) Dinucleotide fold enrichment: dinucleotide contexts significantly over-represented at high-error positions. Points show fold enrichment (observed/background proportion); horizontal bars extend to the lower 95% confidence bound. Manufacturers are distinguished by color. The dashed diagonal line marks significance at *p*_adj_ = 0.05.

To investigate whether specific nucleotide identities or local sequence contexts are associated with elevated error rates, we applied a binomial enrichment test to identify mononucleotides and dinucleotides over-represented among the top 20% highest-error positions in the ssODNs from each manufacturer. Relative to the background base composition of the ssODN, G was significantly over-represented at high-error positions in all manufacturers (Figure 3A, *p*_adj_ < 0.05, fold over-representation between two and three depending on manufacturer); C-containing positions were also over-represented in Manufacturer A, although to a lower degree. To determine if the base preceding the error position during synthesis predicted the occurrence of an error, we examined the frequency of dinucleotides formed by error positions and the base immediately 3′ to them. We found that G-containing error positions tended to be preceded by A or T (Figure 3B, *p*_adj_ < 0.05, fold over-representation between three and four depending on manufacturer); in addition, C was also found to precede a G in Manufacturers B and C, and CT dinucleotides were more than four-fold over-represented in Manufacturer A. These specificities of synthesis error sequence contexts are broadly consistent with previous studies [2,3,4], and indicate that error-prone positions share identifiable sequence features, and that the relationship between sequence context and error rate differs among manufacturers, potentially reflecting differences in synthesis chemistry or post-synthesis processing.

To directly test whether ssODN synthesis errors are propagated into the genome by HDR and exclude that the observed SNEs and deletions in the ssODNs were the result of PCR or sequencing errors, we compared SNE profiles from direct ssODN sequencing with those from amplicon sequencing of edited HSPCs. We reasoned that when an ssODN is used in a gene editing protocol, errors in its sequence will be incorporated into the genome by HDR. However, while SNEs are present throughout the 168 bp ssODN, only those present in the region where the ssODN is used as a template for HDR (HDR region) will be incorporated into the genome. Thus, true ssODN synthesis errors will produce an excess of sequence variants in edited genomic DNA only in the vicinity of the Cas9 cleavage site, where HDR occurs. In contrast, errors that result from the ssODN sequencing process will not be present in the genomic DNA following editing. When ssODNs with high rates of errors are used as a template for HDR, we observe that the errors are incorporated into the genomic DNA of edited cells. We analyzed targeted amplicon sequencing data generated from the region surrounding the SCD mutation in both SCD and healthy donor HSPCs that had been edited with our protocol using ssODNs from the three vendors (Figure 1). Amplicons derived from HBB alleles that were subjected to HDR can be identified by the presence of sequence changes programmed into the ssODN: an alteration in the PAM motif, reversion of the sickle mutation to wild type, and a neutral substitution in the base preceding the sickle mutation that allows identification of HDR in wild type cells [7,8]. The sequenced amplicon encompasses a 350 bp window surrounding the Cas9 cut site and the entire sequence of the ssODN.

**Figure 4:**
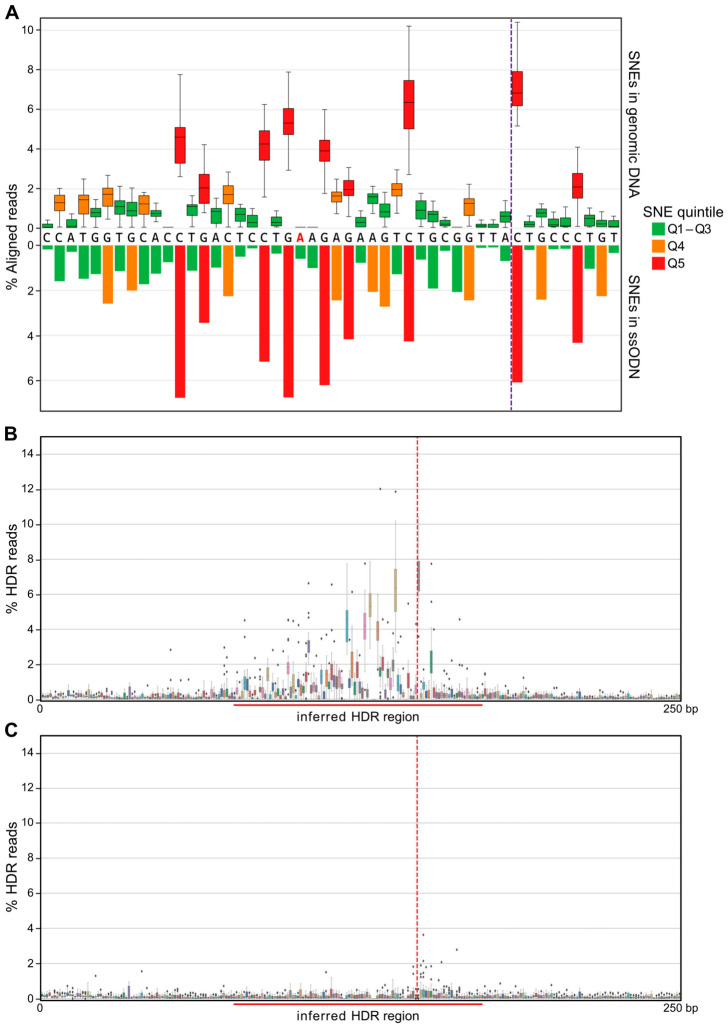
SNE frequency in genomic DNA from HSPCs edited with ssODN from Manufacturer A. (**A**) Comparison of SNEs in ssODN and edited genomic DNA. The *x*-axis shows the region containing the Cas9 cleavage site (marked by the vertical dashed line), the PAM motif, and the SCD mutation. The *y*-axis shows SNE frequency calculated as the percentage of aligned reads that contains an error. Error frequencies are colored by quintile. The position of the SCD mutation is shown by the red character in the sequence. *Upper panel*: SNEs in genomic DNA isolated from multiple edited HSPC samples using ssODN from manufacturer A were quantified and displayed as boxplot. *Lower panel*: SNEs detected by direct sequencing of ssODN from manufacturer A, shown as a reference for template-associated errors. (**B**) SNE frequency in HDR-derived reads from genomic DNA (HDR-derived reads are defined by editing of the PAM site). A 250-base window containing the cleavage site is shown, with the inferred HDR template region labeled. The Cas9 cleavage site is indicated by the vertical dashed red line. The HDR region spans approximately 50–75 bp to the left of the cleavage site to 10 bp to its right. Although the plot encompasses the full 168 bp ssODN, errors are incorporated into a smaller region of ~60–85 bp centered on the cleavage site, consistent with the boundaries of the HDR template. The absence of SNEs outside this region strongly suggests that observed errors derive from the ssODN template. (**C**) In amplicons derived from unedited reads, SNEs were rarely observed within or outside the HDR region. A small excess of SNEs proximal to the cleavage site likely reflects HDR events that initiated at the cleavage site but did not extend to the PAM site, precluding definitive classification as HDR-derived reads. Across all panels the vertical red dashed line marks the Cas9 cleavage site; the horizontal red solid line marks the inferred HDR region, the genomic interval templated by the ssODN and spanning approximately 50 to 75 bp upstream and 10 bp downstream of the cleavage site within which ssODN errors are incorporated.

Figure 4A overlays SNE frequency at each position in the ssODN with SNE frequency in HDR-derived genomic reads from multiple independently edited HSPC samples. Strikingly, high-frequency SNE positions in the ssODN (colored red, indicating the top quintile error rate) correspond to positions with elevated SNE frequency in the genomic amplicons, while positions with low ssODN error rates (green, bottom three quintiles) show correspondingly low SNE frequencies in the genomic DNA. This positional concordance is consistent with direct templating of genomic sequence by the error-containing ssODN during HDR. Furthermore, SNEs are incorporated into the HSPCs at rates that recapitulate the variation by manufacturer seen in the direct ssODN sequencing (Supplementary Figure S3).

SNEs in the three ssODN were consistently observed at the same positions in the amplicon sequencing data at a frequency roughly correlated with SNE frequency in the ssODNs. Outside of the region where the ssODN was used as a template for HDR, we see errors at a rate that would be expected from PCR bias or sequencing errors, and comparable to the rate observed in reads from unedited samples (Figure 4B). Deletions, which occurred more uniformly across the length of the ssODN, were evenly distributed only in the HDR region, and not in the surrounding sequence (Figure S4). Furthermore, the ssODN SNEs were not observed in unmodified reads (alleles that lacked base changes at the PAM and the site of the sickle mutation) (Figure 4C).

The presence of SNEs in the ssODN also provides valuable information on the extent and polarity of HDR in our protocol. We noted that the frequency of SNE propagation by HDR (determined as the frequency of SNEs in the genome amplicon sequence) was consistent with HDR initiating at the Cas9 cleavage site, proceeding largely asymmetrically (to the left of the cleavage site in Figure 4A), and attenuating with distance from the cleavage site. Thus, ssODN SNEs can be used as markers of the HDR conversion tract to define the genomic region where the ssODN was used as a template for HDR at highest frequency: in our system, this was the genomic interval roughly 50–75 bp upstream and 10 bp downstream of the Cas9 cleavage site.

In sequenced alleles that had undergone HDR, rates of incorporation of ssODN synthesis errors into genomic DNA occurred at a frequency as high as 12% for SNEs and up to 3% for deletions at individual positions. Incorporation of SNEs was observed with ssODNs from all three manufacturers at a rate proportional to the SNE frequency in the respective ssODNs (Figure 4A and Supplementary Figure S3). Deletions in the ssODNs were also incorporated into the genomic DNA by HDR, but at a lower frequency than the SNEs (Supplementary Figure S4). Significantly, 90% of the deletions seen were 1 or 2 bp frame-shift deletions: although they occur at a lower frequency than SNEs, they would have a more consequential effect on β-globin production.

This investigation of edited cell products thus corroborates the direct sequencing of ssODNs and highlights the variation in synthesis errors among manufacturers (Figure 2 and Figure S2). The basis of the ssODN synthesis errors was not apparent to any of the manufacturers, all of whom used the standard chemical synthesis by coupling of phosphoramidite analogs and were unaware of the presence of sequence errors in the synthesis products.

## 4. Discussion

Synthesis errors in ssODN templates for HDR-mediated gene editing have implications for both pre-clinical and clinical development. The method we have used improves on previous efforts [2,3,4] by allowing investigators to establish the extent of sequence errors in any ssODN using standard reagents. The enrichment of errors at C and G positions is consistent with known challenges in phosphoramidite-based synthesis at consecutive or isolated G and C residues and may help guide future improvements to synthesis protocols. None of the prior studies determined whether synthesis errors are faithfully propagated into the genome by HDR; here, we demonstrate that SNEs and deletions present in the donor template are incorporated into edited alleles. The quantitative relationship between ssODN SNE frequency and genomic SNE frequency implies that improvements in ssODN synthesis quality will translate directly into reduced error burden in edited alleles. Critically, these errors are invisible to standard QC methods (HPLC, mass spectrometry), which assess overall purity and molecular weight but cannot resolve single-nucleotide compositional errors at specific positions. The data therefore argue that the sequencing approach described here should be a prerequisite for regulatory submissions for clinical gene editing programs, both to characterize the error profile of any given batch and to enable informed risk assessment of the amino acid consequences of the most prevalent synthesis errors.

The vendor-to-vendor differences in error rate and error context are consistent with differences in synthesis and post-synthesis processing. Coupling efficiency at isolated and consecutive G and C positions, the efficiency of the capping step (which prior work links to G-to-A substitutions [3,4]), and purification grade all plausibly contribute, and the error contexts we observe differ by vendor in a manner compatible with differing chemistries. These data do not establish a mechanism: the vendors treat these process parameters as proprietary and did not disclose them and a definitive attribution would require controlled synthesis experiments outside the scope of this study.

The effects of SNEs and deletions on editing outcomes will vary depending on the specific gene and the type of sequence modified: errors may have more impact in coding regions, but the effects of specific errors in transcriptional control elements are less predictable. In the example of our protocol, available clinical data predict that amino acid changes caused by SNEs in the edited region of *HBB* are likely to create benign ß-globin variants, while frameshift deletions are predicted to create deleterious ß-thalassemia-like loss-of-function alleles [10,11,12,13,14]. Introducing a low frequency of loss-of-function alleles into an autologous HSPC product carries consequences distinct from those of the benign substitutions. Even at low frequency, such alleles add to the clonal heterogeneity of the graft: a fraction of corrected cells would carry a non-functional HBB allele alongside the intended correction, which bears on product characterization and on the interpretation of editing outcomes at single-cell resolution. These are risk-assessment predictions based on the amino acid and reading-frame consequences of the observed errors, not phenotypes measured here; the data establish that the underlying alleles are generated at a quantifiable frequency, and the functional predictions follow from established genotype-phenotype relationships.

The generality of these findings has two distinct senses. The synthesis error itself is a property of chemical oligonucleotide synthesis: it depends on vendor and sequence context but is independent of the target locus, the nuclease, and the cell type, so any HDR protocol that uses a chemically synthesized ssODN donor is subject to it. The functional consequence of an incorporated error is, by contrast, locus- and context-dependent, with coding sequence and transcriptional control elements differing in both the predictability and the severity of the outcome. The assay reported here transfers directly to other loci, nucleases, and cell types; the interpretation of any errors it detects must be made locus by locus.

Incorporating ssODN sequencing into clinical manufacturing is practical at the batch level. The assay uses commercially available single-stranded library kits and standard short-read sequencing, requires sub-microgram input, and can be multiplexed across batches, so its per-batch cost and turnaround are modest relative to the cost of clinical ssODN manufacturing and to the downstream risk it characterizes. We therefore frame the recommendation as batch-level sequence characterization integrated into existing release testing rather than as a separate, bespoke workflow.

Several developments could reduce ssODN synthesis errors at their source. Improvements in synthesis chemistry, including the optimization of the capping step and the non-canonical nucleosides shown to suppress G-to-A substitutions [3,4], address the most common substitutions directly; enzymatic or hybridization-based error correction and higher-grade purification lower the error burden of a finished oligonucleotide; and sequence design that avoids the high-risk contexts identified here reduces the probability of an error within the HDR tract. Until synthesis fidelity improves, batch-level deep sequencing provides a near-term means to characterize and bound the error content of each clinical ssODN lot.

## Data Availability

The sequence data used in this study are available in the NCBI SRA archive as BioProject PRJNA1281709.

## Supplementary Materials

The following supporting information can be downloaded at https://www.mdpi.com/article/10.3390/genes17070729/s1, Figure S1: Schematic of analysis pipeline; Figure S2: SNE and indel frequency in the three ssODNs; Figure S3: SNEs in genomic DNA; Figure S4: Deletions in genomic DNA.

## Abbreviations

The following abbreviations are used in this manuscript:

ssODN	single-stranded oligodeoxynucleotide
HDR	homology-directed repair
CRISPR	clustered regularly interspaced short palindromic repeats
Cas9	CRISPR-associated protein 9
DSB	double-stranded break
sgRNA	single guide RNA
HSPC	hematopoietic stem and progenitor cell
SCD	sickle cell disease
HBB	β-globin gene
SNE	single-nucleotide error
indel	insertion or deletion
UMI	unique molecular identifier
PAM	protospacer-adjacent motif
HPLC	high-performance liquid chromatography
SDS-PAGE	sodium dodecyl sulfate-polyacrylamide gel electrophoresis
NHEJ	non-homologous end joining
BH	Benjamini–Hochberg
