# Supplementary material for: Oligonucleotide Synthesis Errors Are a Source of Untoward Variation in HDR-Mediated Gene Editing
Source: Genes (Basel). 2026 Jun 24;17(7):729. doi: 10.3390/genes17070729 (PMC13409629; doi:10.3390/genes17070729)
Supplement: Supplementary file 1 [file genes-17-00729-s001.zip › Figure_S2 SNE and indel frequency in the three ssODNs.pdf]

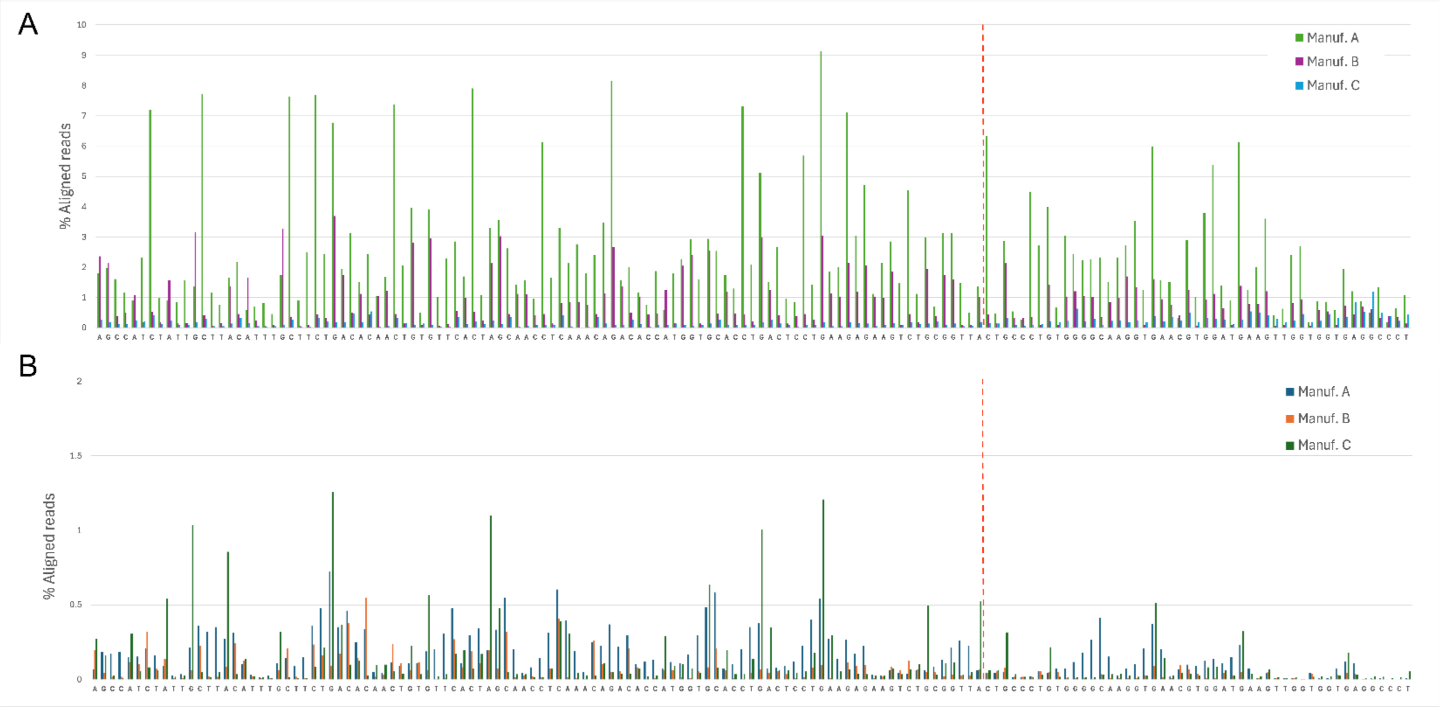

Figure S2. SNE (**A**) and indel (**B**) frequency in the three ssODNs, determined by direct sequencing. The full length ssODN is shown, with 8 bases pruned from each end to eliminate library preparation artifacts that appear at the ends. Cas9 cleavage location is marked by a vertical dashed red line (see Figure 2 for a detailed view of the 50-base region surrounding the Cas 9 cut site). Error frequency (SNE or indel) was calculated as the fraction of aligned reads with an error at a given position.
