# Supplementary material for: Oligonucleotide Synthesis Errors Are a Source of Untoward Variation in HDR-Mediated Gene Editing
Source: Genes (Basel). 2026 Jun 24;17(7):729. doi: 10.3390/genes17070729 (PMC13409629; doi:10.3390/genes17070729)
Supplement: Supplementary file 1 [file genes-17-00729-s001.zip › Figure_S3 SNEs in genomic DNA.pdf]

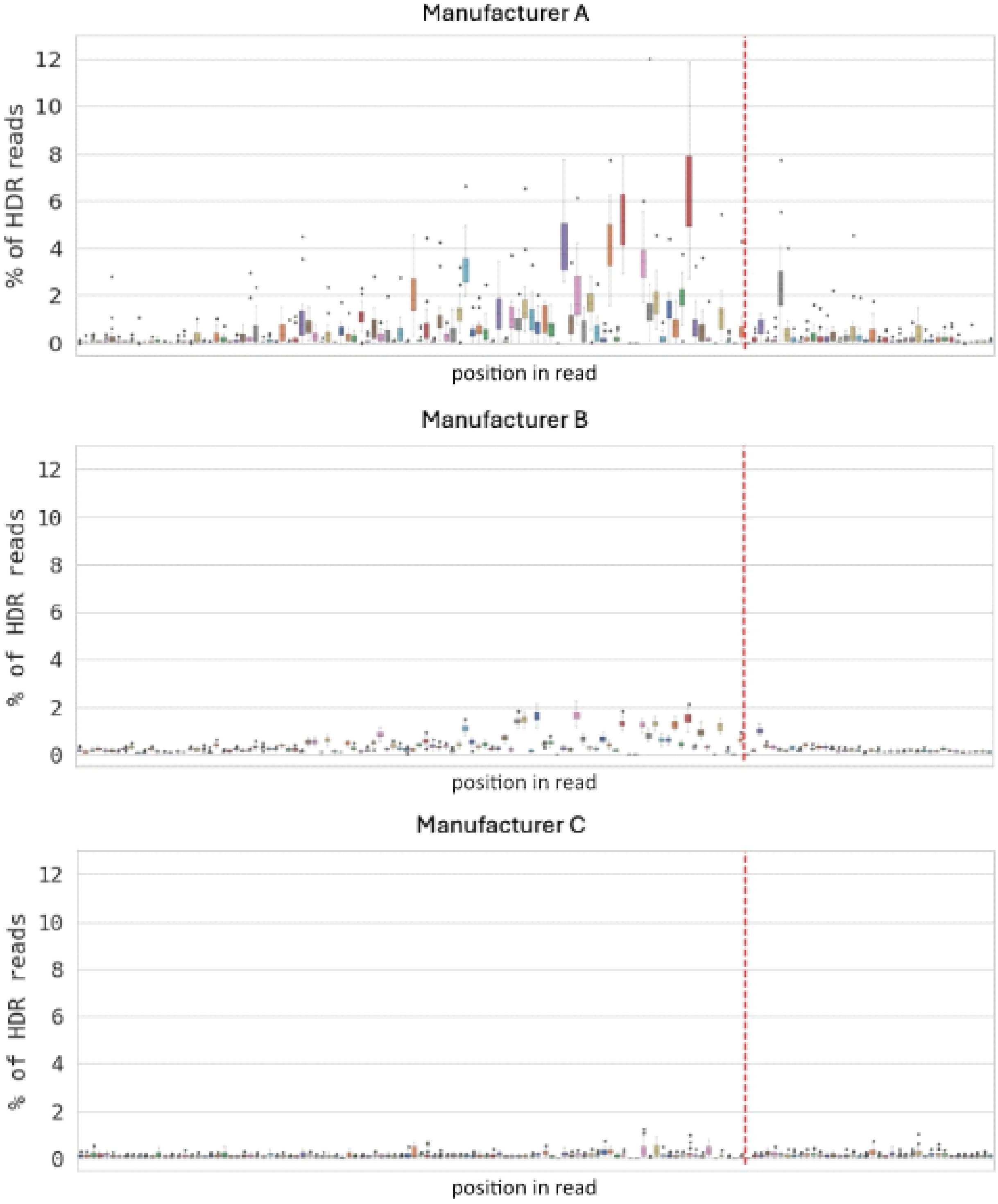

Figure S3: SNEs in genomic DNA from multiple edited HSPC samples using ssODNs from three different manufacturers were quantified as a percent of HDR reads and plotted. The dashed red line represents the Cas9 cleavage site.
