# Supplementary material for: Oligonucleotide Synthesis Errors Are a Source of Untoward Variation in HDR-Mediated Gene Editing
Source: Genes (Basel). 2026 Jun 24;17(7):729. doi: 10.3390/genes17070729 (PMC13409629; doi:10.3390/genes17070729)
Supplement: Supplementary file 1 [file genes-17-00729-s001.zip › Figure_S4 Deletions in genomic DNA.pdf]

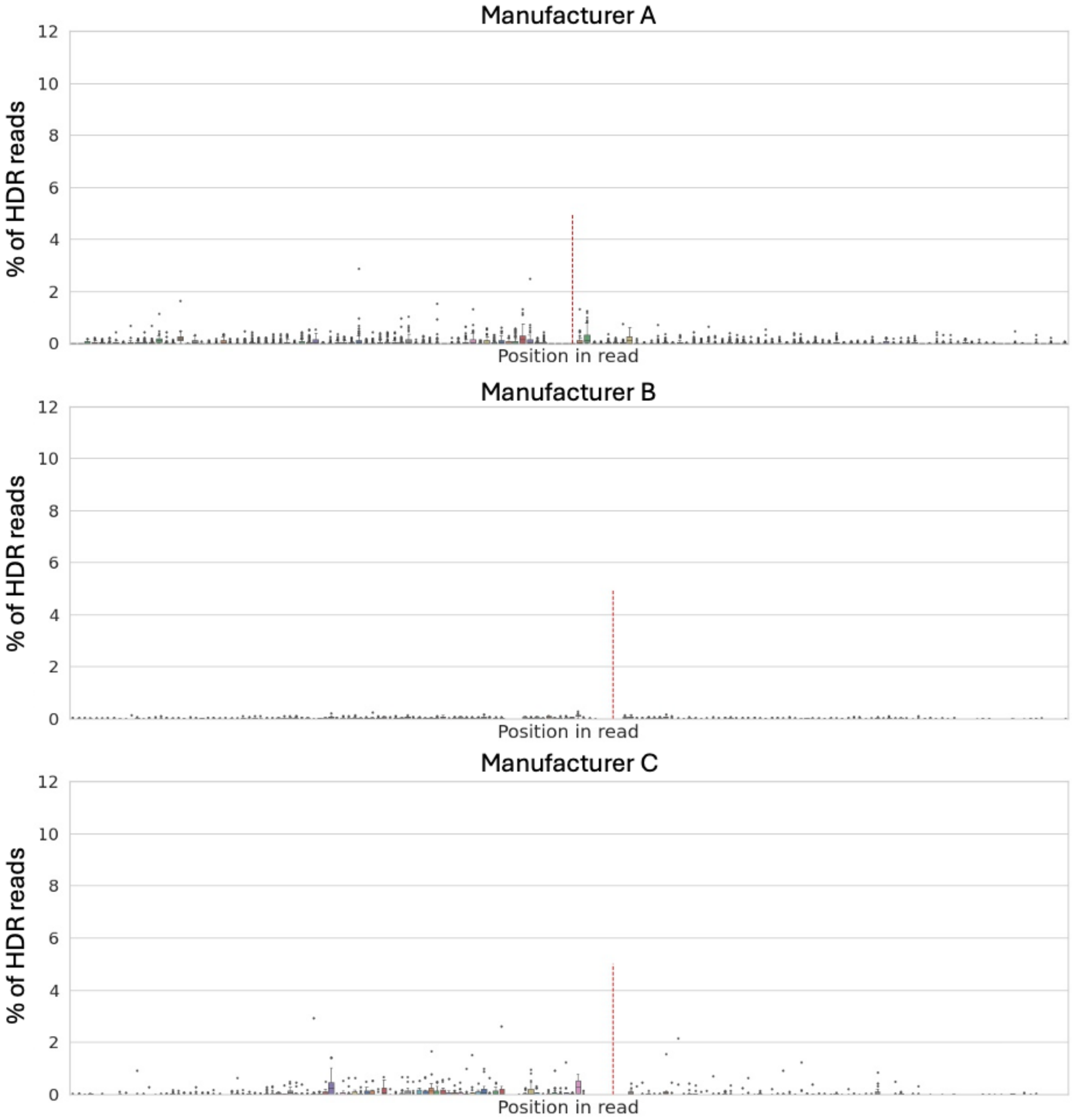

Figure S4: Deletions in genomic DNA from multiple HSPC samples edited using ssODNs from the three different manufacturers, quantified as a percent of HDR reads and plotted. Dashed red line represents cleavage site. Deletions are not seen directly adjacent to the cleavage site because reads with deletions at the cleavage site were counted as NHEJ reads.
