# Supplementary figures and images for: Oligonucleotide Synthesis Errors Are a Source of Untoward Variation in HDR-Mediated Gene Editing
Source: Genes (Basel). 2026 Jun 24;17(7):729. doi: 10.3390/genes17070729 (PMC13409629; doi:10.3390/genes17070729)

### Pipeline for analyzing ssODN sequencing data

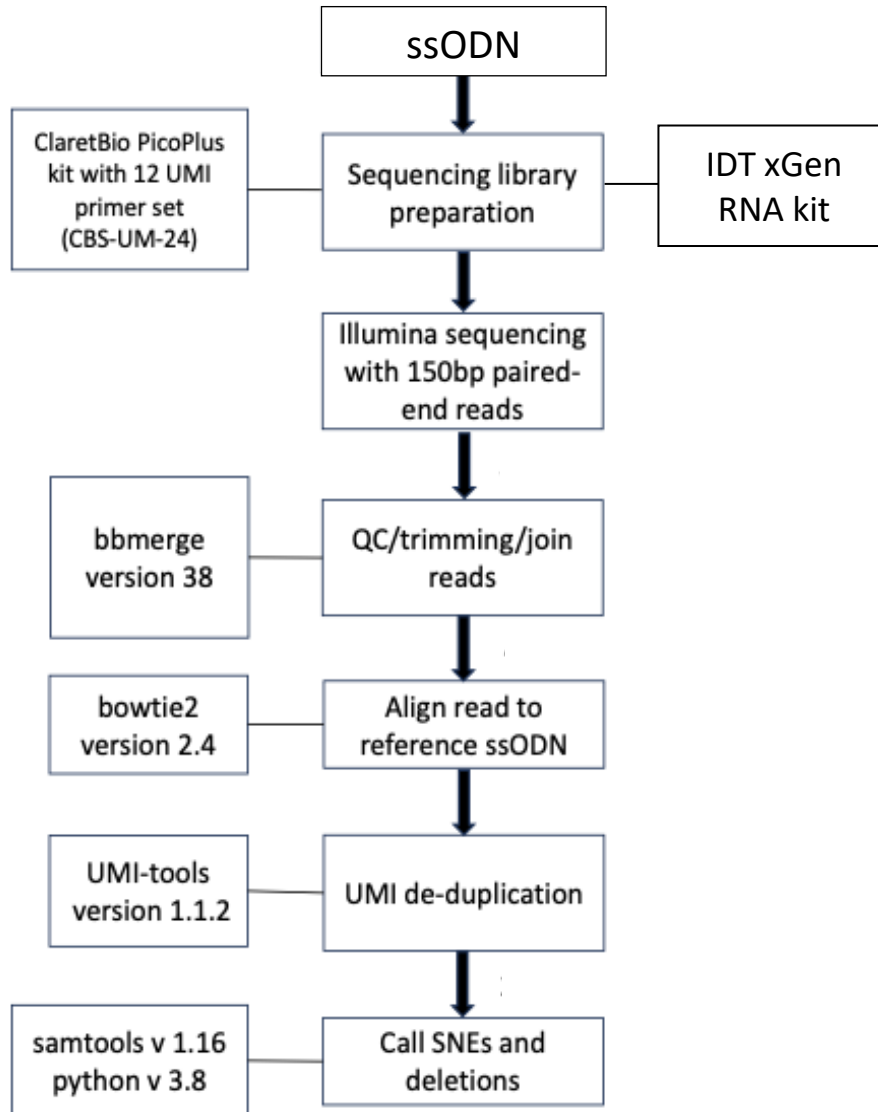

**Figure S1:** Schematic of analysis pipeline.

Supplement: Supplementary file 1 [file genes-17-00729-s001.zip › Figure_S1 Schematic of analysis pipeline.pdf]
